# A systematic review assessing the under-representation of elderly adults in COVID-19 trials

**DOI:** 10.1186/s12877-020-01954-5

**Published:** 2020-12-20

**Authors:** Virginie Prendki, Noam Tau, Tomer Avni, Marco Falcone, Angela Huttner, Laurent Kaiser, Mical Paul, Yaara Leibovici-Weissmann, Dafna Yahav, on behalf of ESCMID Study Group for Infections in the Elderly (ESGIE)

**Affiliations:** 1 Division of Internal Medicine for the Aged, Geneva University Hospitals, and University of Geneva, Hôpital des Trois-Chêne, Chemin du Pont-Bochet 3, 1226 Genève, Thônex Switzerland; 2 Faculty of Medicine, University of Geneva, Geneva, Switzerland; 3 Division of Infectious Diseases, Geneva University Hospitals, Geneva, Switzerland; 4 Department of Diagnostic Imaging, Chaim Sheba Medical Center, Ramat Gan, Israel; 5 Sackler Faculty of Medicine, Tel-Aviv University, Ramat Aviv, Tel Aviv, Israel; 6 Medicine A, Rabin Medical Center, Beilinson Hospital, Petah-Tikva, Israel; 7 Infectious Diseases Unit, Department of Clinical and Experimental Medicine, University of Pisa, Pisa, Italy; 8 Division of Infectious Diseases, Rambam Health Care Campus, Haifa, Israel; 9 Department of Acute Geriatrics, Rabin Medical Center, Beilinson Hospital, Petah Tikva, Israel; 10 Infectious Diseases Unit, Rabin Medical Center, Beilinson Hospital, Petah-Tikva, Israel

**Keywords:** COVID-19, Elderly, Clinical trials, systematic review

## Abstract

**Background:**

Coronavirus disease (COVID-19) has caused a pandemic threatening millions of people worldwide. Yet studies specifically assessing the geriatric population are scarce. We aimed to examine the participation of elderly patients in therapeutic or prophylactic trials on COVID-19.

**Methods:**

In this review, randomized controlled trials (RCTs; *n* = 12) comparing therapeutic or prophylactic interventions registered on preprint repositories and/or published since December 2019 were analyzed. We searched in PubMed, leading journals websites, and preprint repositories for RCTs and large observational studies. We aimed to describe the age of included patients, the presence of an upper age limit and of adjusted analyses on age, any exclusion criteria that could limit participation of elderly adults such as comorbidities, cognitive impairment, limitation of life expectancy; and the assessment of long-term outcomes such as the need of rehabilitation or institutionalization. Mean participant ages were reported and compared with observational studies.

**Results:**

Twelve RCTs assessing drug therapy for COVID-19 were included. Mean age of patients included in RCTs was 56.3 years. An upper age limit was applied in three published trials (25%) and in 200/650 (31%) trials registered at clinicaltrials.gov. One trial reported a subgroup analysis in patients ≥65. Patients were excluded for liver-function abnormalities in eight trials, renal disease in six, cardiac disease or risk of *torsade de pointes* in five, and four for cognitive or mental criteria, which are frequent comorbidities in the oldest patients. Only three trials allowed a family member to provide consent. Patients enrolled in RCTs were on average 20 years younger than those included in large (*n* ≥ 1000) observational studies. Seven studies had as their primary outcome a clinical endpoint, but none reported cognitive, functional or quality of life outcomes or need for rehabilitation or long-term care facility placement.

**Conclusions:**

Elderly patients are clearly underrepresented in RCTs, although they comprise the population hardest hit by the COVID-19 pandemic. Long-term outcomes such as the need of rehabilitation or institutionalization were not reported. Future investigations should target specifically this vulnerable population.

## Background

The novel coronavirus SARS-CoV-2 has caused a pandemic threatening millions of people worldwide. The clinical presentation of coronavirus disease (COVID-19) varies widely from asymptomatic carriage to pneumonia, acute respiratory distress syndrome and septic shock [1, 2]. The highest mortality is observed in the elderly [3, 4]. The Chinese Center for Disease Control and Prevention (CDC) report that, of 72,314 hospitalized and ambulatory patients with COVID-19, 81% had mild disease. The case fatality rate was 14.8 and 8% among patients aged ≥80 years and 70–79 years, respectively [4]. In March 2020, 4,226 COVID-19 cases were reported to the US CDC; 31% were 65 years or older, and 45% of hospitalizations, 53% of intensive care unit (ICU) admissions and 80% of deaths occurred in patients ≥65 years. The worst outcomes occurred among patients aged ≥85 years [5]. The International Severe Acute Respiratory and Emerging Infections Consortium (ISARIC) database reported 26,276 patients hospitalized with COVID-19: 14,193 of them (70%) were aged ≥60 and 92% of the 5,358 deaths occurred in these patients [6].

Many drugs are being actively studied as therapy for or prophylaxis against COVID-19, with over 1,500 studies currently registered at the clinicaltrials.gov registry. Yet studies specifically assessing the geriatric population are scarce [7–10]. Avni et al. showed that elderly patients were often excluded from randomized clinical trials evaluating agents for bacterial pneumonia [11]. An intervention may differ in both its effectiveness and safety when applied to an elderly population as opposed to younger populations. Hence we aimed to examine the participation of elderly patients in therapeutic or prophylactic trials on COVID-19. Our objectives were to assess whether they were explicitly or indirectly excluded from clinical research.

## Methods

### Study design

In this review, we included all randomized clinical trials (RCTs) registered on preprint repositories and/or published between December 2019 and May 22nd 2020. Participants in the trials were adult patients (age ≥ 18 years) with COVID-19. The review was performed in accordance with PRISMA guidelines [12]. Clinical trials comparing any pharmacologic agents and devices to another intervention or control in patients with COVID-19 were included. COVID-19 diagnosis was considered as defined in individual trials.

The following interventions were considered for inclusion: drug therapy/prophylaxis, biological therapy (e.g., immunoglobulins, convalescent plasma), device (e.g., continuous positive airway pressure, oxygen therapy). Phase I trials and studies evaluating diagnostic tests were excluded, as were studies including only pediatric patients. We also excluded trials assessing Chinese traditional medicine, as these are less relevant for other countries. For comparison, we also searched for large observational cohorts including COVID-19 patients. We planned to extract data from cohorts including over 1,000 patients and reporting outcomes. We aimed to compare the age of included patients to those included in RCTs.

### Search strategy

We searched for full-text RCTs in PubMed, leading internal medicine and infectious diseases journals (see list below), and preprint repositories (including medRxiv at https://www.medrxiv.org/; arxiv at https://arxiv.org/; and bioRxiv at https://www.biorxiv.org/). Search terms used for PubMed search were “COVID19 OR COVID-19 OR SARS-CoV-2 OR 2019-nCoV OR SARS2”, combined with the Cochrane filter for RCTs [13]. For preprint repositories, the search term “COVID-19” was combined with “randomized”.

Journal sites searched included those of *The New England Journal of Medicine* (https://www.nejm.org/coronavirus), *The Lancet* (https://www.thelancet.com/coronavirus), *JAMA* (https://jamanetwork.com/journals/jama/pages/coronavirus-alert), *Annals of Internal Medicine* (https://annals.org/aim/pages/coronavirus-content), the journals of the Infectious Diseases Society of America (IDSA) (https://www.idsociety.org/public-health/COVID-19-Resource-Center/), Emerging Infectious Diseases journal (at https://wwwnc.cdc.gov/eid/), and *Clinical Microbiology and Infection* (https://www.clinicalmicrobiologyandinfection.com/). We hand searched all study titles published in “COVID-19 resource centers” of each of the above journals for relevant RCTs or observational studies. We also searched for unpublished RCTs in the clinicaltrials.gov registry, using their link to listed clinical studies related to the coronavirus disease (COVID-19).

An additional search for observational studies was performed using the term “COVID19 OR COVID-19 OR SARS-CoV-2 OR 2019-nCoV OR SARS2”, combined with ‘observational’ OR ‘cohort’ OR ‘prospective’ OR ‘retrospective’. We also reviewed observational data from the ISARIC database [6]. No language restrictions were applied to any of the searches. Two reviewers independently conducted the search and applied inclusion criteria (either VP, NT, or YLW). Any discrepancies were resolved by a third reviewer (DY). The titles and as needed, abstracts or full texts, of the studies were each reviewed by the authors for their relevance. Specifically for observational studies, we also applied a criterion of sample size of over 1000 patients.

### Data extraction

Two reviewers independently extracted the following data. For full-text RCTs: publication status and site, setting (hospital vs. other), purpose (prophylaxis vs. treatment), inclusion and exclusion criteria with emphasis on criteria that may eventually lead to exclusion of elderly: any upper age limit, comorbidities, or exclusion for medications/polypharmacy or due to mental or cognitive disorders. Similarly, consent options, potentially a factor limiting elderly patients’ participation, number with limited life expectancy or do-not-resuscitate orders, were also documented. Additional data extracted included interventions and outcomes, including report of outcomes for age-specific subgroups. Studies were also analyzed according to their primary endpoint, i.e., clinical (mortality, time to clinical improvement or clinical improvement rate, duration of invasive mechanical ventilation, hospitalization, transfer to/from intensive care unit (ICU), discharge to long-term-care facilities (LTCF) or rehabilitation, adverse events (AEs), including serious AEs and QTc > 500 milliseconds) or virologic outcomes (duration of viral detection in clinical samples). For observational studies, we extracted data regarding patients’ age and mortality.

### Data analysis

Inclusion and exclusion criteria were analyzed, with emphasis on age and other confounding factors cited above. The percentage of trials performing age-adjusted analyses was also reported. Combined mean age for all RCTs was calculated. For studies reporting age as median, the median was considered equivalent to the mean, as suggested by the Cochrane handbook [13]. Mean participant ages were compared between RCTs and large observational studies. For RCTs, risk of bias was assessed using the domains recommended by the Cochrane handbook. These were graded as low, high or unknown risk of bias, according to the Cochrane handbook’s criteria [13]. For observational studies we used NIH Study Quality Assessment Tool for observational cohort and cross-sectional studies (https://www.nhlbi.nih.gov/health-topics/study-quality-assessment-tools). Two authors performed the quality assessment independently (DY and NT).

## Results

### Published RCTs

**Fig. 1:**
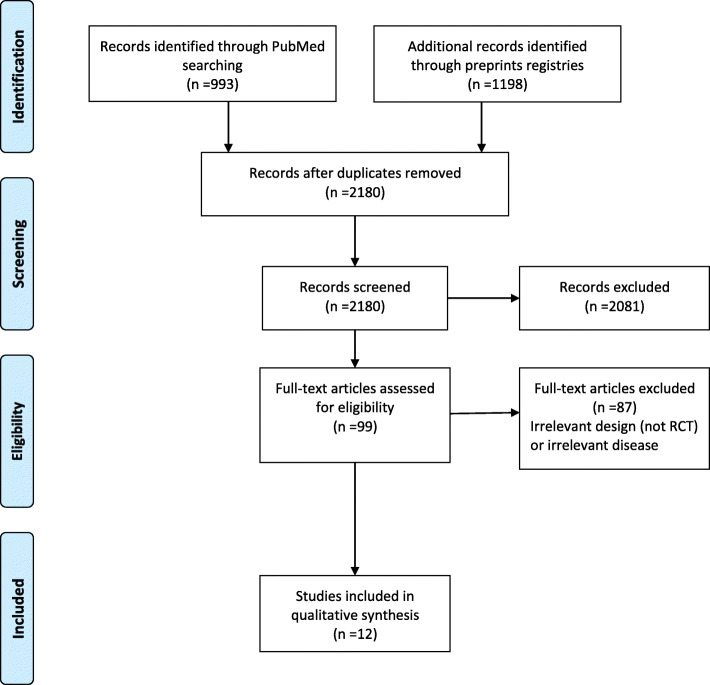
Flow of the Search Procedure for Randomized Controlled Trials

The search yielded 2191 RCTs; after applying eligibility criteria, 12 full-text trials assessing various treatment options for COVID-19 were included (Fig. 1). Risk of bias assessment of these trials is detailed in Supplementary Table 1. All trials but one were judged low risk of bias for allocation generation; eight for allocation concealment; and four were double blind studies. (For full data see Supplementary Table 1). All trials addressed drug therapy, of which eight were performed in China. Table 1 summarizes characteristics of included trials. Two were published in PubMed [14, 15], four at journal websites [16–19] and six in medRxiv [19–24]. Four evaluated lopinavir/ritonavir-based therapy [14, 17, 19, 24], three chloroquine or hydroxychloroquine therapy [15, 20, 23], two favipiravir or baloxavir [21–23], two remdesivir [16, 18] and one α-lipoic-acid [25]. The mean age of patients included in RCTs was 56.3 years. No trial specifically targeted the elderly. An upper age limit was reported in three studies (25%). Chen et al. reported 60/240 patients aged ≥65 years (25%) [20], Beigel 382/1063 (36%) patients aged ≥65 years [18], and Zhong nine patients between 60 and 70 and two > 70 years among a total of 17 patients (53%) [25]. Three studies performed age-specific subgroup analyses, among which one for patients ≥65 [23] (Table 1). Eight, six and five trials had exclusion criteria of liver function abnormalities or severe liver disease [14, 16, 19–24], severe kidney disease [16, 19, 20, 22–24], and heart disease/arrhythmia/prolonged QT, respectively [17, 19, 20, 23, 24]. No study excluded patients due to polypharmacy but three excluded patients taking medication potentially interacting with lopinavir/ritonavir, ribavirin, interferon or arbidol [14, 17, 24]. Four trials excluded patients due to the presence of a mental or cognitive criteria: “a mental illness affecting therapeutic compliance” [24], “unable to cooperate with investigators due to cognitive impairment or poor mental status” [23], “inability to comprehend and to follow all required study procedures” [17, 18]. One study used an exclusion criterion of patients who would probably not cooperate or finish the study: “base on the researcher’s judgment, there are other factors that may cause the subject to be forced to terminate the study midway, such as other serious diseases, serious laboratory examination abnormalities, other factors that affect the safety of the subject or study data and blood sample collection” [22]. Five trials excluded patients for expected survival time under 48 h or for critical illness [20–22, 24, 25]. Only six trials allowed consent to be provided by a legal guardian [14–18, 21], and three reported allowing a family member to provide consent [6, 15, 17]. Primary outcomes varied: only seven RCTs chose a clinical outcome (one reported 28-day mortality and six clinical improvement or recovery); five RCTs reported virological outcomes (time to or rate of negative polymerase chain reaction (PCR)). None of these studies reported on discharge to rehabilitation or long-term care facilities (LTCF) or other clinical outcomes relevant for the geriatric population, such as cognitive or functional decline.

**Table 1:** Characteristics of included randomized controlled trials

Study ID	Intervention	Publication site	Country	Disease severity included (most patients)	Number randomized	Primary outcome	Age	Upper age limit inclusion	Number. age ≥ 65 y	Sub-group of elderly reported	Exclusion by mental or cognitive disorder	Consent options
Cao 2020 [14]	LPV/r	PubMed	China	Severe	199	Time to clinical improvement	58.0 (49.0–68.0)	NS	NS	NS	NS	Patient/legal guardian
Borba 2020 [15]	High-dose CQ	PubMed	Brazil	Severe	81	Mortality (28 day)	51.1 (13.9)	NS	NS	NS	NS	Patient/legal guardian/family
Wang 2020 [16]	Remdesivir	Journal site (Lancet)	China	Severe	237	Time to clinical improvement	65 (56–71)	NS	NS	NS	NS	Patient/legal guardian
Hung 2020 [17]	LPV/r based combinations	Journal site (Lancet)	Hong Kong	Mild/moderate	127	Time to negative RT-PCR	52 (32–62)	NS	NS	NS	Inability to comprehend and to follow all required study procedures	Patient/legal guardian/family
Beigel 2020 [18]	LPV/r based combinations	Journal site (Lancet Infectious Diseases)	China	Mild/moderate	101	Time to negative RT-PCR	42.5 (11.5)	65	NS	For safety - 18-40 vs 41–65	NS	NS (“Written informed consent”)
Zeng 2020 [19]	Remdesivir	Journal site (NEJM)	Multinational (mainly USA)	Severe	1063	Time to recovery	58.9 (15.0)	NS	382	For age ≥ 65	Understands planned study procedures	Patient/legal guardian
Chen Z 2020 [20]	Favipiravir vs arbidol	medRxiv	China	Moderate	240	Clinical recovery rate at 7 days	NS	NS	60/240	NS	NS	NS (“Informed consent was obtained from all patients”)
Chen C 2020 [21]	HCQ	medRxiv	China	Mild	62	Time to clinical recovery	44.7 (15.3)	NS	NS	NS	NS	Patient/legal guardian
Lou 2020 [22]	baloxavir marboxil vs favipiravir	medRxiv	China	NS	30	Rate of RT-PCR negativity at 14 days and time to clinical improvement	52.5 (12.5)	85	NS	NS		NS (“Consent was obtained”)
Tang 2020 [23]	HCQ	medRxiv	China	Mild/moderate	150	Rate of RT-PCR negativity at 28 days	46.1 (14.7)	NS	72/150 age ≥ 45	For primary outcome age > =45 vs < 45	Unable to cooperate with investigators due to cognitive impairment or poor mental status	NS (“Written informed consent was obtained from all patients”)
Li 2020 [24]	LPV/r vs arbidol	medRxiv	China	Mild/moderate	86	Time to negative RT-PCR	49.4 (14.7)	80	NS	NS	Mental illnesses affecting treatment compliance	NS (“Written informed consent was obtained from all screened patients”)
Zhong 2020 [25]	α-Lipoic acid	medRxiv	China	Critical	17	SOFA Score	63 (59–66)	NS	9/17 age 60–70, 2/17 age > 70	NS	NS	NS (“Patients who cannot sign informed consent must obtain informed consent from the independent authorized nurse”)

*LPV/r* Lopinavir–Ritonavir, *HCQ* Hydroxychloroquine, *CQ* chloroquine, *NS* non-specified, *RT-PCR* Reverse-Transcription-Polymerase Chain Reaction

### Ongoing RCTs

Our clinicaltrials.gov search yielded 1651 registered studies addressing COVID-19; among these, 870 interventional studies included only adults, and 650 were RCTs evaluating therapeutic and prophylactic interventions for COVID-19. Two hundred RCTs (200/650, 31%) had an upper age limit: 56 used a cutoff of 65 years, 153 of 80 years, and 47 of 99 years.

### Observational studies

**Fig. 2:**
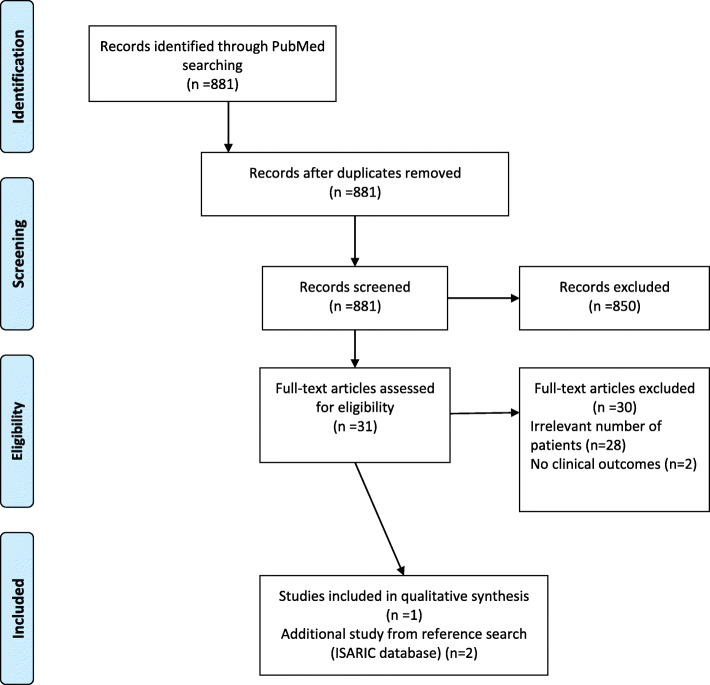
Flow of the Search Procedure for Observational Studies

We reviewed 881 titles from PubMed searching for observational studies including over 1000 patients and reporting clinical outcomes (Fig. 2). We found two such cohorts, from which we extracted data on age and mortality: Guan et al. (China) described 1099 COVID-19 patients hospitalized in 552 hospitals [26]. Median age was 47 (IQR 35–58). Of the entire cohort, only 15.1% were 65 or older. When stratified according to disease severity, patients ≥65 years old made up 27% of severe patients but only 12.9% of non-severe patients. Overall mortality was 1.4%; it was not reported by age group. Richardson et al. (USA) reported the outcomes of 5700 patients hospitalized with COVID-19 in the New York City area [27]. Here the median age was 63 (IQR 52–75, range 0–107); 36% were ≥ 70 years old. Overall mortality was 9.7% (2634 were discharged or had died at the study’s end of follow-up). In the 60–69, 70–79, 80–89 and ≥ 90-year age groups, mortality was 6.4, 12.6, 24.9 and 28.6%, respectively. Mortality rates for those receiving mechanical ventilation in the 18-to-65 and > 65-year age groups were 76.4 and 97.2%, respectively. In the same age groups, mortality rates for those not requiring mechanical ventilation were 19.8 and 26.6%, respectively. Quality rating of these two studies according to NIH quality assessment tool was judged as good quality for Richardson et al. [27] and fair quality for Guan et al. [26] (Supplemental Table 2).

As of May 6th 2020, the ISARIC database contained information on 20,276 COVID-19 patients from 35 countries [6]. Median age was 72 (range 0–104 years). Here, 53% of the patients were ≥ 70 years old. Overall mortality was 26.6%, yet in the 60–69 and ≥ 70-year age groups, it was 21 and 39%, respectively.

## Discussion

Here we have assessed the inclusion, planned and actual, of elderly patients in COVID-19 trials on pharmacologic agents and devices. We show that elderly persons are underrepresented and demonstrate that no trial has specifically addressed them. Only one reported clinical outcomes in subgroup analyses of this population, the most frequently and severely affected by the disease [18]. Three of 12 published RCTs actively imposed an upper age limit on participants, as did 200 of 650 interventional trials identified at clinicaltrials.gov.

Some trials essentially impose a functional upper age limit even when no age criterion is explicitly stated. Of the published RCTs, four (42%) excluded patients due to cognitive capacity, eight (67%) due to liver disease, six (50%) because of severe kidney disease and five (42%) because of heart disease or risk of *torsade de pointes*. These are all conditions that are significantly more prevalent in patients aged ≥65 years.

Patients included in published RCTs had an average age of 56.3 years: they were younger than those included in observational studies [6, 27]. This difference calls into question the RCTs’ external validity, and should remind clinicians to exercise prudence when considering data from such trials for clinical decision-making in the elderly. The majority of RCTs were conducted in China. According to the Wuhan Aging Working Committee Office, by the end of 2019, 14.7% of the population in Wuhan county was ≥65 years, and 2.9% were 80 or older [28]. Interestingly, according to the United States Census Bureau, on April 2020, 14.1% of persons in New-York were aged ≥65 years. This suggests that cultural, rather than demographic, factors might be driving the younger ages observed in the Chinese trials discussed here.

There are understandable and sound reasons for the exclusion of elderly patients from some trials, particularly those designed for the early development of novel therapeutics. There is often limited experience in elderly populations with the drug of interest [29]. These patients have an increased risk of drug-drug interactions due to potential polypharmacy and age-related physiological changes affecting pharmacokinetics and pharmacodynamics. Remdesivir is contraindicated in patients with creatinine clearance < 30 mL/min and elevated liver function tests [30, 31]. Hydroxychloroquine and lopinavir/ritonavir are known to cause QT prolongation with a possible increased risk of *torsades de pointes* [32].

Yet when drugs of interest are being given off-label to elderly patients essentially *en masse*, trial protocols should adapt to reflect the larger clinical reality around them, allowing for increased and more equitable representation of this population. Indeed, increased mortality was observed among hospitalized US veterans treated with hydroxychloroquine for COVID-19. This finding highlights the urgent need for RCTs expressly targeting the group most affected by COVID—and most likely to receive the drug off-label anyway [33]. In a similar fashion, Avni et al. showed that elderly patients were often excluded from RCTs assessing bacterial pneumonia and reported that the participants were significantly younger than in observational studies [11]. This serves to remind us the underrepresentation of elderly in RCTs in general [34]. The obvious under-representation of the elderly in COVID-19 trials is an acute manifestation of a larger problem: the elderly tend to be disproportionately excluded from RCTs in all domains. Elderly patients with cognitive, psychiatric or physical comorbidities are largely absent from the RCT “record”, leaving clinicians to rely on data from inferior studies such as retrospective case series and cohorts, which are notoriously unreliable due to confounding by indication and other biases [35].

As the aging population continues to grow in size, medical research must better reflect this growing segment of the population. This is especially true regarding COVID-19, which is more common and more severe in elderly, causing devastating effects in nursing homes and LTCFs. Conducting clinical trials in elderly adults should compel the clinician to choose relevant outcomes; when planning an RCT, one must ask: “what is important to the elderly patient?” Such outcomes should include immediate but also long-term outcomes such as deterioration of cognitive and functional capacity, quality-of-life and the need for rehabilitation or LTCF placement. We found no study reporting or intending to report such outcomes. It is well established that severe infections have long-term consequences that continue well beyond the first month [36]. Rahmel at al showed that patients surviving sepsis had a better 5-year survival when benefiting from a rehabilitation program [37]. In an RCT of 72 COVID-19 patients (median age 69), Liu et al. reported that a six-week respiratory rehabilitation program could improve pulmonary function and quality of life [38]. Finally, ethical standards should facilitate inclusion of elderly adults with more adapted informed consent, including the possibility to obtain consent by proxy if the patient has diminished capacity. It should be noted that since older patients are at risk of severe disease, concerns are raised regarding their inclusion in placebo-controlled trials. Nevertheless, some suggest that any COVID-19 patient should be enrolled in a well-designed trial in order to achieve proven treatments for the diseases [39]. Hence, trials including elderly should be carefully planned, with attention to the special characteristics of elderly and specific safety issues.

### Strengths and limitations

Multiple data sources were comprehensively reviewed in this search, using a broad search term of all COVID-19 associated studies. However, for the search of observational studies a significant hand search was required, which may have resulted in omissions. In addition, the search of leading journals was based on the journals’ COVID-19 resource centers, without a specific search term used by this review authors.

### Conclusion

Elderly persons are underrepresented in COVID-19 RCTs, although they are the demographic most frequently and severely affected by the disease. Clinical research including the elderly has never been easy; nevertheless, future trials will need to address this vulnerable and oft-forgotten population, particularly when these individuals are regularly receiving off-label therapies anyway. Both interventional RCTs, including elderly patients, are needed; as well as observational studies including both older and non-older patients, the latter to clarify the special characteristics of older patients with COVID-19.

## Supplementary Information

**Additional file 1: Supplementary Table 1.** Quality assessment – included randomized controlled trials

**Additional file 2: Supplementary Table 2.** Quality assessment – included observational studies

## Data Availability

The datasets generated during and/or analysed during the current study are available from the corresponding author on reasonable request.

## Abbreviations

AE: Adverse events
CDC: Center for Disease Control and Prevention
COVID-19: Coronavirus disease
ICU: Intensive care unit
IDSA: Infectious Diseases Society of America
ISARIC: International Severe Acute Respiratory and Emerging Infections Consortium
LTCF: Long-term-care facilities
PCR: Polymerase chain reaction
RCT: Randomized controlled trials
SARS-CoV-2: Severe acute respiratory syndrome coronavirus 2
