# Supplementary material for: A systematic review assessing the under-representation of elderly adults in COVID-19 trials
Source: BMC Geriatr. 2020 Dec 20;20:538. doi: 10.1186/s12877-020-01954-5 (PMC7749979; doi:10.1186/s12877-020-01954-5)
Supplement: Supplementary file 1 — Additional file 1: Supplementary Table 1. Quality assessment – included randomized controlled trials [file 12877_2020_1954_MOESM1_ESM.docx]

**Supplementary Table 1 – Quality assessment – included randomized controlled trials**

| **Study ID** | **Allocation generation** | **Allocation concealment** | **Blinding** | **Incomplete outcome data** | **Selective outcome reporting** | **Other bias** |
| --- | --- | --- | --- | --- | --- | --- |
| Cao 2020 ^14^ | Low | Low | Open | Low | Low | No |
| Borba 2020 ^15^ | Low | Low | Double blind | Low | High | No |
| Wang 2020 ^16^ | Low | Low | Double blind | Low | Low | Yes (early stop) |
| Hung 2020 ^17^ | Low | Unclear | Open | Low | Low | No |
| Chen YK 2020 ^26^ | Low | Unclear | Open | Low | Low | No |
| Beigel 2020 ^19^ | Unclear | Unclear | Double blind | Low | Low | No |
| Chen Z 2020 ^20^ | Low | Unclear | Double blind | Low | Low | No |
| Chen C 2020 ^21^ | Low | Low | Open | Low | Low | No |
| Tang 2020 ^23^ | Low | Low | Open | Low | Low | Yes (early stop) |
| Lou 2020 ^22^ | Low | Low | Open | Low | Low | No |
| Li 2020 ^24^ | Low | Low | Patient and outcome assessor blinded | Low | Low | No |
| Zhong 2020 ^25^ | Low | Low | Outcome assessor blinded | Low | Low | No |
