# Supplementary material for: A systematic review assessing the under-representation of elderly adults in COVID-19 trials
Source: BMC Geriatr. 2020 Dec 20;20:538. doi: 10.1186/s12877-020-01954-5 (PMC7749979; doi:10.1186/s12877-020-01954-5)
Supplement: Supplementary file 2 — Additional file 2: Supplementary Table 2. Quality assessment – included observational studies [file 12877_2020_1954_MOESM2_ESM.docx]

**Supplementary Table 2 – Quality assessment – included observational studies**

|  | Guan et al. ^27^ | | Richardson et al. ^28^ | |
| --- | --- | --- | --- | --- |
| **Criteria** | **Reviewer 1** | **Reviewer 2** | **Reviewer 1** | **Reviewer 2** |
| 1. Research question | Yes | Yes | Yes | Yes |
| 2. Study population a | Yes | Yes | Yes | Yes |
| 3. Study population b | No | No | Yes | Yes |
| 4. Groups recruited | Yes | Yes | Yes | Yes |
| 5. Sample size justification | No | No | No | Yes |
| 6. Exposure prior to outcome | Yes | Yes | Yes | Yes |
| 7. Timeframe | No | No | No | No |
| 8. Exposure amount | NA | NA | NA | NA |
| 9. Exposure measures | Yes | Yes | Yes | Yes |
| 10. Exposure repeated test | No | No | Yes | Yes |
| 11. Outcomes | Yes | Yes | Yes | Yes |
| 12. Outcome assessors blinded | No | No | No | No |
| 13. Loss to follow up | Yes | Yes | Yes | Yes |
| 14. Confounding variables | Yes | Yes | Yes | No |
| **Quality Rating** | Fair | Fair | Good | Good |
